# *β*-empirical Bayes inference and model diagnosis of microarray data

**DOI:** 10.1186/1471-2105-13-135

**Published:** 2012-06-19

**Authors:** Mohammad Manir Hossain Mollah, M Nurul Haque Mollah, Hirohisa Kishino

**Affiliations:** 1 Graduate School of Agricultural and Life Sciences, The University of Tokyo, 1-1-1 Yayoi, Bunkyo-ku, Tokyo 113-8657, Japan; 2 Department of Statistics, University of Rajshahi, Rajshahi-6205, Bangladesh

## Abstract

**Background:**

Microarray data enables the high-throughput survey of mRNA expression profiles at the genomic level; however, the data presents a challenging statistical problem because of the large number of transcripts with small sample sizes that are obtained. To reduce the dimensionality, various Bayesian or empirical Bayes hierarchical models have been developed. However, because of the complexity of the microarray data, no model can explain the data fully. It is generally difficult to scrutinize the irregular patterns of expression that are not expected by the usual statistical gene by gene models.

**Results:**

As an extension of empirical Bayes (EB) procedures, we have developed the *β*-empirical Bayes (*β*-EB) approach based on a *β*-likelihood measure which can be regarded as an ’evidence-based’ weighted (quasi-) likelihood inference. The weight of a transcript *t* is described as a power function of its likelihood, *f*^*β*^(***y***_*t*_|***θ***). Genes with low likelihoods have unexpected expression patterns and low weights. By assigning low weights to outliers, the inference becomes robust. The value of *β*, which controls the balance between the robustness and efficiency, is selected by maximizing the predictive *β*_0_-likelihood by cross-validation. The proposed *β*-EB approach identified six significant (*p*<10^−5^) contaminated transcripts as differentially expressed (DE) in normal/tumor tissues from the head and neck of cancer patients. These six genes were all confirmed to be related to cancer; they were not identified as DE genes by the classical EB approach. When applied to the eQTL analysis of *Arabidopsis thaliana*, the proposed *β*-EB approach identified some potential master regulators that were missed by the EB approach.

**Conclusions:**

The simulation data and real gene expression data showed that the proposed *β*-EB method was robust against outliers. The distribution of the weights was used to scrutinize the irregular patterns of expression and diagnose the model statistically. When *β*-weights outside the range of the predicted distribution were observed, a detailed inspection of the data was carried out. The *β*-weights described here can be applied to other likelihood-based statistical models for diagnosis, and may serve as a useful tool for transcriptome and proteome studies.

## Background

Microarray technology has made it possible to investigate the expression levels of thousands of genes simultaneously. At the same time, it presents a challenging statistical problem because of the large number of transcripts with small sample sizes that are surveyed. A fundamental statistical problem in microarray gene expression data analysis is the need to reduce the dimensionality of the transcripts. A common approach for dimensionality reduction is the identification of differentially expressed (DE) genes under different conditions or groups. By associating differential expressions with the genotypes of molecular markers, useful information on the regulatory network can be obtained [1-4]. By assigning DE genes to the list of gene sets, it is possible to obtain a useful biological interpretation [5,6]. Further, because the number of DE genes that influence a certain phenotype may be large while their relative proportion is usually small, it is challenging to identify these DE genes from among the large number of recorded genes [7-14]. Two main types of statistical inferences for the identification of DE genes have been used: (1) classical parametric (for example, *t*-test, *F*-test, likelihood ratio test) and non-parametric [13,15-18] procedures; and (2) empirical Bayes (EB) parametric [8-12,14,19-22] and non-parametric [23,24] procedures. In general, classical procedures detect the DE genes using p-values (significance levels) either estimated by permutation or based on the distribution of a test statistic, while EB procedures use the posterior probability of differential expression for the identification of DE genes.

Classical parametric testing procedures (like the t-, F- or *χ*^2^-test) may produce misleading results when they are used directly to determine DE genes, because these methods strongly depend on the sample size and normality of the expression data [2,17,25-28]. EB hierarchical models have gradually become more popular than classical methods for identification of DE genes because these models explicitly specify the distribution of the gene-specific mean expression levels and the distribution of the expression profiles around the means. EB approaches detect a DE gene by sharing information across the whole genome; such approaches also work well for small sample sizes. A popular EB approach using a hierarchical gamma-gamma (GG) model [11] was developed for the identification of DE genes. The model was extended [8] to replicate chips with multiple conditions and a new option of using a hierarchical lognormal-normal (LNN) model was introduced. The GG and LNN models were both developed under the assumption of a constant coefficient of variation across genes. However, this assumption is not very realistic and it can negatively affect the resulting inference. To overcome these problems, both models were extended assuming gene-specific variances [29]. It has been shown that the extended versions of both the GG and LNN models outperform previous versions of GG and LNN [8,11] as well as the nonparametric SAM (significance analysis of microarray) model [17]. A different version of the extended EB-LNN model that assumes gene-specific variances [30] is also available. The performance of the EB-LNN model has been investigated using several normalization techniques [1]. Most of the algorithms described above are not robust against outliers. Some recent studies have reported that the assumption of normality does not hold for most of the existing microarray data [31,32]. One of the causes for the breakdown of the normality assumption for gene expression data may be data contamination by outliers. The cDNA microarray data are often contaminated by outliers that arise because of the many steps that are involved in the experimental process from hybridization to image analysis. A few Bayesian parametric approaches [32-35] for the robust identification of DE genes are available; however, the identification of contaminating genes or irregular patterns of expression has never been discussed. When one of these Bayesian parametric approaches is used, it is difficult to scrutinize or diagnose contaminating DE genes in reduced gene expression datasets. As a result, any further statistical investigations like, for example, the clustering/classification of the genes in the reduced gene expression dataset may produce misleading results.

To overcome this problem, we developed a *β*-empirical Bayes (*β*-EB) approach as an extension of the EB-LNN model [8,30] assuming gene-specific variances for the identification of DE genes. The *β*-EB model is a unique parametric approach because, not only is it robust against outliers, but it also detects contaminating genes and statistically diagnoses gene expression profiles. These features may significantly improve any further statistical analysis of gene expression data like clustering/classification. The *β*-EB method was developed based on the *β*-divergence estimation that was proposed by Basu et al. [36] and fully described later by Minami and Eguchi [37]. It was shown that the minimization of *β*-divergence is equivalent to maximizing the weighted (quasi-) likelihood which we have called *β*-likelihood. The proposed *β*-EB method introduces a *β*-weight function that produces smaller weights for contaminating genes and larger weights for uncontaminating genes to obtain weighted estimates for the model parameters. Thus, based on the value of the *β*-weight function, the inference becomes robust. The value of *β*, which controls the balance between robustness and efficiency, is selected by maximizing the predictive *β*_0_-likelihood. When the dataset satisfies the model assumptions and does not include outliers, *β* may be selected to be 0. On the other hand, when the model is misspecified or when the data include outliers, the selected *β* may be positive.

Here, we introduce the *β*-weight distribution as a sensor that detects outliers or the misspecification of the model. When *β*-weights outside the range of the predicted distribution are observed, a detailed inspection of the data is conducted. Microarray data offers a unique opportunity to investigate the distribution of the *β*-weights because the data represents the expression of a large number of genes. By contracting the observed distribution of the weights with the predicted distribution, it is possible to detect outliers and to diagnose the hierarchical model statistically. Although, in this paper, we have introduced a Gaussian model, the *β*-likelihood-based approach could still be applied for robustification of any likelihood-based estimation of statistical models and this feature may serve as a useful tool for genome data analysis.

## Methods

Here the extension of the EB-LNN model assuming gene-specific variances [8,30] by *β*-divergence, which we have called the *β*-EB approach, for the identification of DE genes, is discussed. The simulated and real microarray gene expression datasets that we have analyzed to investigate the performance of the proposed method are also described.

### Empirical Bayes hierarchical model

If the transcript-specific parameter $\theta_{t}=(\mu_{t},\theta_{t}^{*})$, where *μ*_*t*_ and $\theta_{t}^{*}$ are the location and scale parameters respectively, then the conditional likelihood of the *t*th transcript’s expression measurement ***y***_*t*_=(*y*_*t*1_,*y*_*t*2_,…,*y*_*tn*_) can be expressed as $\overset{n}{\underset{i=1}{\prod }}f_{\mathrm{obs}}\left(y_{\mathrm{ti}}|\theta_{t}\right)$ (*t*=1,2,…,*T*). The location parameter *μ*_*t*_ follows the prior distribution, *Π*(*μ*_*t*_|***θ***), where ***θ***is the hyper-parameter specifying the prior distribution. The predictive likelihood of ***y***_*t*_ (unconditional on the location parameter *μ*_*t*_) is obtained by integrating over the location parameter, *μ*_*t*_, as follows:

(1) $$f_{0}(y_{t}|\theta ,\theta_{t}^{*})=\int \left(\overset{n}{\underset{i=1}{\prod }}f_{\mathrm{obs}}\left(y_{\mathrm{ti}}|\mu_{t},\overset{*}{\underset{t}{\theta }}\right)\Pi (\mu_{t}|\theta )\right)d\mu_{t}.$$

When expression measurements between two groups (for example, different cell types) are compared for transcript *t*, the measurements are partitioned into two user defined groups *G*_1_ and *G*_2_ of sizes *n*_1_ and *n*_2_ respectively, where *n*_1_ + *n*_2_=*n*. If there is no significant difference between the means of the two groups, the gene is assumed to be equivalently expressed (EE); otherwise, it is assumed to be a DE gene. If the *t*th transcript is DE, the two groups will have different mean expression levels, $\mu_{t}^{\left(j\right)},\;j=1,2$. Given the values of $\mu_{t}^{\left(j\right)},\;j=1,2$ and $\theta_{t}^{*}$, the conditional likelihood of $y_{t}=\left(y_{t}^{\left(1\right)}:y_{t}^{\left(2\right)}\right)$ is written as follows:

(2) $$\begin{array}{cc} f_{1}(y_{t}|\mu_{t}^{\left(1\right)},\mu_{t}^{\left(2\right)},\theta_{t}^{*}) & =\left(\overset{n_{1}}{\underset{i=1}{\prod }}f_{\mathrm{obs}}\left(y_{\mathrm{ti}}|\mu_{t}^{\left(1\right)},\theta_{t}^{*}\right)\right)\\ \;\times \left(\overset{n_{2}}{\underset{i^{\prime \prime }=1}{\prod }}f_{\mathrm{obs}}\left(y_{ti^{\prime \prime }}|\mu_{t}^{\left(2\right)},\theta_{t}^{*}\right)\right), \end{array}$$

because components of ***y***_*t*_are independent of each other. Assuming that the group means $\mu_{t}^{\left(j\right)},\;j=1,2$ (such that $\mu_{t}^{\left(1\right)}\neq \mu_{t}^{\left(2\right)}$) independently originate from *Π*(*μ*_*t*_|***θ***), then the predictive likelihood of ***y***_*t*_ (unconditional on the location parameters $\mu_{t}^{\left(j\right)},\;j=1,2$) is obtained as a mean of the conditional likelihood of ***y***_*t*_(2) over the prior distribution of $\mu_{t}^{\left(1\right)}$ and $\mu_{t}^{\left(2\right)}$ as follows:

(3) $$\begin{array}{lll} \;f_{1}(y_{t}|\theta ,\theta_{t}^{*}) & =\int \int f_{1}(y_{t}|\overset{\left(1\right)}{\underset{t}{\mu }},\overset{\left(2\right)}{\underset{t}{\mu }},\overset{*}{\underset{t}{\theta }})\Pi (\overset{\left(1\right)}{\underset{t}{\mu }}|\theta )\Pi (\overset{\left(2\right)}{\underset{t}{\mu }}|\theta )\; & \;\\ & \;\times d\mu_{t}^{\left(1\right)}d\mu_{t}^{\left(2\right)}\; & \;\\ & =\left(\;\int \;\left(\overset{n_{1}}{\underset{i=1}{\prod }}f_{\mathrm{obs}}\;\left(\;y_{\mathrm{ti}}|\overset{\left(1\right)}{\underset{t}{\mu }},\overset{*}{\underset{t}{\theta }}\right)\right)\Pi \left(\;\;\overset{\left(1\right)}{\underset{t}{\mu }}|\theta \;\right)d\overset{\left(1\right)}{\underset{t}{\mu }}\right)\; & \;\\ & \;\times \;\left(\;\int \;\;\;\left(\overset{n_{2}}{\underset{i^{\prime \prime }=1}{\prod }}f_{\mathrm{obs}}\left(\;y_{ti^{\prime \prime }}|\overset{\left(2\right)}{\underset{t}{\mu }},\overset{*}{\underset{t}{\theta }}\;\right)\;\;\right)\;\Pi \;\left(\;\overset{\left(2\right)}{\underset{t}{\mu }}|\theta \;\right)\;d\overset{\left(2\right)}{\underset{t}{\mu }}\;\right)\; & \;\\ \;\; & =\;\;f_{0}(y_{t}^{\left(1\right)}|\theta ,\theta_{t}^{*})f_{0}(y_{t}^{\left(2\right)}|\theta ,\theta_{t}^{*}).\; \end{array}$$

Because it is unknown whether the *t*th gene is EE or DE between the two groups, the final likelihood of ***y***_*t*_(unconditional on the location parameters) becomes a mixture of two distributions (1) and (3) as follows:

(4) $$f(y_{t}|\theta ,\theta_{t}^{*},p_{0})=p_{0}f_{0}(y_{t}|\theta ,\theta_{t}^{*})+p_{1}f_{1}(y_{t}|\theta ,\theta_{t}^{*}).$$

Here, *p*_0_and *p*_1_ are the mixing proportions of the EE and DE transcripts in the two user defined groups respectively, such that *p*_0_ + *p*_1_=1. The posterior probability of differential expression (PPDE) is calculated by Bayes rule using the estimates of *p*_0_, *f*_0_ and *f*_1_ as follows:

(5) $$\frac{p_{1}f_{1}(y_{t}|\theta ,\theta_{t}^{*})}{p_{0}f_{0}(y_{t}|\theta ,\theta_{t}^{*})+p_{1}f_{1}(y_{t}|\theta ,\theta_{t}^{*})}.$$

It should be noted here that ***θ***and $\theta_{t}^{*}$ in equations (1)-(5) are assumed to be exactly the same.

### Maximum *β*-likelihood estimation of mixture distribution using an EM-like algorithm to calculate *β*-posterior probabilities of differential expressions

Box and Cox [38] proposed a family of power transformations of the dependent variable in regression analysis to robustify the normality assumption. By choosing an appropriate value of *λ* in the transformation,

(6) $$\begin{array}{lll} g_{\lambda }(y) & =\left\{\begin{array}{ll} \frac{y^{\lambda }-1}{\lambda } & \left(\lambda >0\right)\\ \log y & (\lambda =0), \end{array}\right)\; & \; \end{array}$$

the standard linear regression model with the normality assumption fits well to a wide range of data. Inspired by this idea, Basu *et al*[36] and Minami and Eguchi [37] proposed a robust and efficient method for estimating model parameter ***θ*** by minimizing a density power divergence in a general framework of statistical modeling and inference. They [36,37] have also shown that minimizer of density power divergence is equivalent to the maximizer of *β*-likelihood function. According to the current problem in this paper, the *β*-likelihood function for ***θ*** given the values of the mixing parameter *p*_0_=1−*p*_1_ and the gene specific scale parameter $\theta_{t}^{*}$ for all *t* can be written as

(7) $$L_{\beta }(\theta |y)=\frac{1}{\mathrm{T\beta}}\overset{T}{\underset{t=1}{\sum }}f^{\beta }(y_{t}|\theta ,\theta_{t}^{*},p_{0})-l_{\beta }(\theta ),$$

where *f*(.) is the mixture of distributions as defined in (4) and $l_{\beta }(\theta )=\frac{1}{1+\beta }\int f^{\beta +1}(y|\theta ,\theta_{t}^{*},p_{0})dy-\frac{\beta -1}{\beta }$ which is independent of observations. Because the gradient of (6) can be converted as follows,

(8) $$\begin{array}{lll} \;\frac{\partial }{\partial \theta }L_{\beta }(\theta |y) & =\frac{1}{T}\;\overset{T}{\underset{t=1}{\sum }}f^{\beta }(y_{t}|\theta ,\theta_{t}^{*},p_{0})\frac{\partial }{\partial \theta }\log\left(f(y_{t}|\theta ,\theta_{t}^{*},p_{0})\right)\; & \;\\ & \;-\frac{\partial }{\partial \theta }l_{\beta }(\theta ),\; \end{array}$$

the maximum *β*-likelihood estimator (*β*-MLE) of ***θ*** can be regarded as a weighted (quasi-) likelihood estimator. Then the weight of gene *t* is described as a power function of its likelihood, $f^{\beta }(y_{t}|\theta ,\theta_{t}^{*},p_{0})$, where *f*(.) is defined by equation (4). Thus, the genes with low likelihoods have unexpected expression patterns and have low weights because the normal density function produces smaller outputs for larger inputs. By assigning low weights to outliers, the inference becomes robust. It is obvious from (7) that *β*-MLE reduces to the classical MLE for *β*=0. Because the expression pattern (EE or DE) of each gene is unknown, it is difficult to optimize both the classical log-likelihood function and the proposed *β*-likelihood function for directly estimating ***θ***. To overcome this problem, we consider the EM-like algorithm to obtain *β*-MLE of ***θ***treating the mixture distribution (4) as an incomplete-data density. The hyper-parameters ***θ*** and the mixing proportion *p*_0_are estimated by EM algorithm as follows:

The hyperparameters, ***θ****p*_0_ are estimated by the EM algorithm in two steps. **E-step**: Compute the Q-function which is defined by the conditional expectation of the complete-data *β*-likelihood with respect to the conditional distribution of missing data (***Z***) given the observed data (***Y***) and the current estimated parameter value $\theta_{\beta }^{\left(j\right)}$ as follows:

(9) $$\;Q_{\beta }\left(\theta |\theta_{\beta }^{\left(j\right)}\right)=\frac{1}{\mathrm{T\beta}}\overset{T}{\underset{t=1}{\sum }}\overset{1}{\underset{k=0}{\sum }}{\left[p_{k}f_{k}(y_{t}|\theta ,{\hat{\theta }}_{t}^{*})\right]}^{\beta }\;\;\times \pi_{\mathrm{tk}}^{\left(j\right)}-\lambda_{\beta }(\theta )$$

where *k* = 0 for ***y***_*t*_ belongs to EE pattern and *k* = 1 for ***y***_*t*_ belongs to DE pattern. Here

(10) $$\lambda_{\beta }(\theta )=\frac{1}{1+\beta }\int \overset{1}{\underset{k=0}{\sum }}{\left[p_{k}f_{k}(y|\theta ,{\hat{\theta }}^{*})\right]}^{1+\beta }\mathrm{dy}-\frac{\beta -1}{\beta }$$

which does not depend on observations,

(11) $$\pi_{\mathrm{tk}}^{\left(j\right)}=\frac{p_{k}^{\left(j\right)}f_{k}(y_{t}|\theta_{\beta }^{\left(j\right)},{\hat{\theta }}_{t}^{*})}{\overset{1}{\underset{k^{\prime \prime }=0}{\sum }}p_{k^{\prime \prime }}^{\left(j\right)}f_{k^{\prime \prime }}(y_{t}|\theta_{\beta }^{\left(j\right)},{\hat{\theta }}_{t}^{*})},\;(k=0,1)$$

is the posterior probability of *k*th pattern for gene *t* and the value of *p*_1_=1−*p*_0_ is updated by a separate EM formulation as follows:

(12) $$\begin{array}{lll} \;p_{1}^{\left(j+1\right)} & ={\left[\;{\left(\frac{\overset{T}{\underset{t=1}{\sum }}f_{1}^{\beta }(y_{t}|\theta_{\beta }^{\left(j\right)},{\hat{\theta }}_{t}^{*})\pi_{t1}^{\left(j\right)}}{\overset{T}{\underset{t=1}{\sum }}f_{0}^{\beta }(y_{t}|\theta_{\beta }^{\left(j\right)},{\hat{\theta }}_{t}^{*})\pi_{t0}^{\left(j\right)}}\right)}^{\frac{1}{\beta -1}}\;\;+1\;\right]}^{-1}\;\;\;\;\;\;\;,\;\text{for}\;\beta \;>\;0\;\\ & =\frac{1}{T}\overset{T}{\underset{t=1}{\sum }}\pi_{t1}^{\left(j\right)},\;\;\text{for}\;\;\beta =0.\; & \; \end{array}$$

For β→0, the proposed Q-function $Q_{\beta }(\theta |\theta^{\left(j\right)})$ reduces to the standard Q-function *Q*(***θ***|***θ***^(*j*)^) of the standard empirical Bayes approaches [8,30].

**M-step**: Find ***θ***^(*j* + 1)^by maximizing the proposed Q-function as defined in (8). Continue EM iterations up to the convergence of successive estimates of ***θ***. The estimate of ***θ*** after convergence is taken to be the *β*-MLE of ***θ***according to the EM properties.

The tuning parameter, *β*, controls the balance between the robustness and efficiency of the estimators. By setting a tentative value for *β*_0_, the optimal value is estimated by maximizing the predictive *β*_0_-likelihood via a five-fold cross validation. The dataset is divided into five subsets by transcripts. For each value of *β*, the predictive *β*_0_-likelihood of each subset is calculated based on the maximum *β*-likelihood estimates of the parameters based on the rest of the data. Finally, the *β* value that maximizes the average predictive *β*_0_-likelihood is selected as the optimal value of *β*. For more information about *β*-selection, please see [39,40].

Then, based on the estimate values of the model parameters, we can compute the PPDE between two groups of ***y***_*t*_ using equation (5) for all *t*. However, PPDE of contaminated gene using equation (5) might be produced misleading result, since PPDE of ***y***_*t*_ depends on the estimate values of parameters and measurements of ***y***_*t*_. To overcome this problem, we detect contaminated genes using *β*-weight function and replace the contaminated measurements in ***y***_*t*_by its group means. Then we compute the PPDE of contaminated ***y***_*t*_ using equation (5) also. The PPDE based on *β*-MLE, we call *β*-PPDE in this paper. The detail discussion for computation of *β*-PPDE under LNN model is discussed below in the LNN model.

### The LNN model

In this paper, we use the LNN (log-normal-normal) hierarchical model for computing the posterior probability of differential expressions. In the LNN model, log-transformed gene expression measurements are assumed to follow normal distribution for each gene with the transcript-specific parameter $\theta_{t}=(\mu_{t},\theta_{t}^{*})$, where *μ*_*t*_ is the transcript-specific mean and $\theta_{t}^{*}=\sigma_{t}^{2}$ is the transcript-specific variance for gene *t*[8,30]. A conjugate prior for *μ*_*t*_ is assumed to follow the normal with some underlying mean *μ*_0_and variance $\tau_{0}^{2}$; that is, $\Pi \left(\mu_{t}|\theta \right)∼N(\mu_{0},\tau_{0}^{2})$, where $\theta =(\mu_{0},\tau_{0}^{2})$. By integrating as in (1), the density *f*_0_(·) for an *n*-dimensional input becomes Gaussian with the mean vector ***μ***_0_ = ${\left(\mu_{0},\mu_{0},\ldots ,\mu_{0}\right)}^{t}$ and an exchangeable covariance matrix as follows:

(13) $$\Sigma_{\mathrm{tn}}=(\sigma_{t}^{2})I_{n}+(\tau_{0}^{2})M_{n},$$

where ***I***_*n*_ is an *n*×*n* identity matrix and ***M***_*n*_is a matrix of ones.

The gene specific variance $\sigma_{t}^{2}$ is computed separately assuming prior distribution for $\sigma_{t}^{2}$ as scale-inverse $\chi^{2}(\nu_{*},\sigma_{*}^{2})$, where *ν*_∗_ is the degrees of freedom and $\sigma_{*}^{2}$ is the scaled parameter. Yang et al. [30] proposed that $\sigma_{t}^{2}$ could be estimated by a Bayes estimator defined as,

(14) $${\hat{\sigma }}_{t}^{2}=\frac{{\hat{\nu }}_{*}{\hat{\sigma }}_{*}^{2}+(n_{1}+n_{2}-2){\tilde{\sigma }}_{t}^{2}}{n_{1}+n_{2}+{\hat{\nu }}_{*}-2}$$

where

(15) $${\tilde{\sigma }}_{t}^{2}=\frac{(n_{1}-1){\tilde{\sigma }}_{t1}^{2}+(n_{2}-1){\tilde{\sigma }}_{t2}^{2}}{n_{1}+n_{2}-2}$$

is the pooled sample variances with

(16) $${\tilde{\sigma }}_{\mathrm{tg}}^{2}=\overset{n_{g}}{\underset{i=1}{\sum }}{\left(y_{\mathrm{ti}}^{\left(g\right)}-{\bar{y}}_{t}^{\left(g\right)}\right)}^{2}/(n_{g}-1)$$

as the sample variance in group *g*=1,2. By viewing the pooled sample variances ${\tilde{\sigma }}_{t}^{2}$ as a random sample from the prior distribution of $\sigma_{t}^{2}$, the estimates $\left({\hat{\nu }}_{*},{\hat{\sigma }}_{*}^{2}\right)$ of $\left(\nu_{*},\sigma_{*}^{2}\right)$ are obtained using the method of moments. However, it is obvious that (12) will be very sensitive to outliers. Therefore, we have used a maximum *β*-likelihood estimation of $\sigma_{\mathrm{tg}}^{2}$ which is highly robust against outliers [39] and can be obtained iteratively as follows:

(17) $$\begin{array}{lll} \mu_{\mathrm{tg}}^{\left(j+1\right)} & =\frac{\overset{n_{g}}{\underset{i=1}{\sum }}\psi_{\beta }(y_{\mathrm{ti}}^{\left(g\right)}|\mu_{\mathrm{tg}}^{\left(j\right)},{\sigma_{\mathrm{tg}}^{2}}^{\left(j\right)})y_{\mathrm{ti}}^{\left(g\right)}}{\overset{n_{g}}{\underset{i=1}{\sum }}\psi_{\beta }(y_{\mathrm{ti}}^{\left(g\right)}|\mu_{\mathrm{tg}}^{\left(j\right)},{\sigma_{\mathrm{tg}}^{2}}^{\left(j\right)})}\;\\ {\sigma_{\mathrm{tg}}^{2}}^{\left(j+1\right)} & =\frac{\overset{n_{g}}{\underset{i=1}{\sum }}\psi_{\beta }(y_{\mathrm{ti}}^{\left(g\right)}|\mu_{\mathrm{tg}}^{\left(j\right)},{\sigma_{\mathrm{tg}}^{2}}^{\left(j\right)}){\left(y_{\mathrm{ti}}^{\left(g\right)}-\mu_{\mathrm{tg}}^{\left(j\right)}\right)}^{2}}{\overset{n_{g}}{\underset{i=1}{\sum }}\psi_{\beta }(y_{\mathrm{ti}}^{\left(g\right)}|\mu_{\mathrm{tg}}^{\left(j\right)},{\sigma_{\mathrm{tg}}^{2}}^{\left(j\right)})}\; & \; \end{array}$$

where

(18) $$\psi_{\beta }(y_{\mathrm{ti}}^{\left(g\right)}|\mu_{\mathrm{tg}},\sigma_{\mathrm{tg}}^{2})=\exp\left\{-\frac{\beta }{2}{\left(\frac{\overset{\left(g\right)}{\underset{\mathrm{ti}}{y}}-\mu_{\mathrm{tg}}}{\sigma_{\mathrm{tg}}}\right)}^{2}\right\}$$

is the *β*-weight function for estimating robust mean and variance which produces an almost zero or very small weight for *y*_*ti*_ if it is an outlying/extreme observation.

To estimate the hyper-parameters $\theta =(\mu_{0},\tau_{0}^{2})$ by maximizing of the proposed Q-function (8) in the M-step, we compute the gradient of $Q_{\beta }(\theta |\theta^{\left(j\right)})$ with respect to ***θ*** which is given by

(19) $$\begin{array}{cc} \frac{\partial }{\partial \theta }Q_{\beta }(\theta |\theta^{\left(j\right)}) & =\frac{1}{T}\overset{T}{\underset{t=1}{\sum }}\overset{1}{\underset{k=0}{\sum }}{\left[p_{k}f_{k}(y_{t}|\theta ,{\hat{\sigma }}_{t}^{2})\right]}^{\beta }\\ \;\times \frac{\partial }{\partial \theta }\log\left[p_{k}f_{k}(y_{t}|\theta ,{\hat{\sigma }}_{t}^{2})\right]\\ \;\times \pi_{\mathrm{tk}}^{\left(j\right)}-\frac{\partial }{\partial \theta }\lambda_{\beta }(\theta ). \end{array}$$

It reduces to the gradient of the standard Q-function denoted by $\frac{\partial }{\partial \theta }Q(\theta |\theta^{\left(j\right)})$ based on the log-likelihood function for *β*=0. The second term on the right-hand side of equation (15) is independent of observations; the first term is the weighted gradient of *Q*(***θ***|***θ***^(*j*)^) with the weight function ${\left[p_{k}f_{k}(y_{t}|\theta ,{\hat{\sigma }}_{t}^{2})\right]}^{\beta }$. This weight function produces a smaller weight if the *t*th gene is contaminated by outliers; otherwise, it produces a comparatively larger weight for the *t*th gene independent of whether it is EE (*k*=0) or DE (*k*=1). Therefore contaminated genes cannot influence the estimates and robust estimates of the parameters can be obtained. For convenience of choosing the threshold weight to identify contaminated genes statistically, we define the *β*-weight function for the gene *t* as follows

(20) $$\phi_{\beta }(y_{t}|\hat{\theta },{\hat{\sigma }}_{t}^{2},k)\propto {\left[p_{k}f_{k}(y_{t}|\hat{\theta },{\hat{\sigma }}_{t}^{2})\right]}^{\beta },$$

where the circumflex above a parameter indicates the proposed estimate of the parameters. Excluding the normalization constant, the *β*-weight function corresponding to an EE gene becomes,

(21) $$\phi_{\beta }(y_{t}|\hat{\theta },{\hat{\sigma }}_{t}^{2},k=0)=\exp\{-\frac{\beta }{2}{\left(y_{t}-{\hat{\mu }}_{0}\right)}^{\prime \prime }{\hat{\Sigma }}_{\mathrm{tn}}^{-1}(y_{t}-{\hat{\mu }}_{0})\},$$

which measures the deviation of each gene expression data vector from the grand mean vector for the expression of all the genes in the dataset. The *β*-weight function corresponding to a DE gene becomes

(22) $$\begin{array}{lll} \;\phi_{\beta }\left(y_{t}|\hat{\theta },{\hat{\sigma }}_{t}^{2},k=1\right) & =\exp\left[-\frac{\beta }{2}\left\{{\left(\overset{\left(1\right)}{\underset{t}{y}}-{\hat{\mu }}_{0}^{\left(1\right)}\right)}^{\prime \prime }\right)\right)\; & \;\\ & \;\times {\hat{\Sigma }}_{tn_{1}}^{-1}\left(y_{t}^{\left(1\right)}\;-{\hat{\mu }}_{0}^{\left(1\right)}\right)\;+\;{\left(y_{t}^{\left(2\right)}\;-{\hat{\mu }}_{0}^{\left(2\right)}\;\right)}^{\prime \prime }\;\; & \;\\ & \;\times \left(\left({\hat{\Sigma }}_{tn_{2}}^{-1}\left(y_{t}^{\left(2\right)}-{\hat{\mu }}_{0}^{\left(2\right)}\right)\;\right\}\right],\; \end{array}$$

where ${\hat{\mu }}_{0}^{\left(1\right)}$=${\left({\hat{\mu }}_{0},{\hat{\mu }}_{0},\ldots ,{\hat{\mu }}_{0}\right)}^{t}$ and ${\hat{\mu }}_{0}^{\left(2\right)}$=${\left({\hat{\mu }}_{0},{\hat{\mu }}_{0},\ldots ,{\hat{\mu }}_{0}\right)}^{t}$ are the grand mean vectors, and ${\hat{\Sigma }}_{tn_{1}}=({\hat{\sigma }}_{t}^{2})I_{n_{1}}+$$(\tau_{0}^{2})M_{n_{1}}$ and ${\hat{\Sigma }}_{tn_{2}}=({\hat{\sigma }}_{t}^{2})I_{n_{2}}+(\tau_{0}^{2})M_{n_{2}}$ are the exchangeable covariance matrices in two user defined groups. Both the *β*-weight functions defined by equations (17) and (18) for genes *t*=1,2,…,*T*produce weights that are between 0 and 1 for any data vector ***y***_*t*_.

Because, both weight functions are the negative exponential function of the squared Mahalanobis Distance (MD) defined by ${\mathrm{MD}}_{t}={\left(y_{t}-{\hat{\mu }}_{0}\right)}^{\prime \prime }{\hat{\Sigma }}^{-1}(y_{t}-{\hat{\mu }}_{0})\geq 0$ between the data vector ***y***_*t*_ and and the mean vector ${\hat{\mu }}_{0}$. From equations (17) and (18), the *β*-weight for gene *t* decreases when MD_*t*_ increases and increases when MD_*t*_ decreases. That is, the *β*-weight for a gene *t* becomes smaller (≥0) when ***y***_*t*_ is contaminated by outliers, and larger (≤1) when it is not contaminated.

The large number of transcripts in microarray data enables a statistical investigation of the observed distribution of the *β*-weights compared to the predicted distribution under the assumption that the model is correct and the data is free from outliers. To investigate this further, we start with the case where the predicted distribution can be obtained theoretically. When the normality assumptions hold and there are no outliers, and when the gene-specific variance is known for EE genes, the cumulative distribution of the *β*-weight $w_{t}=\phi_{\beta }(y_{t}|\theta ,\sigma_{t}^{2},k=0)$ for gene *t* with known gene specific variance ($\sigma_{t}^{2}$) becomes,

(23) $$\begin{array}{lll} \;G_{t}(w_{0}) & =\mathrm{Pr}\{w_{t}\leq w_{0}\}\; & \;\\ & =\mathrm{Pr}\left\{\exp\left[-\frac{\beta }{2}{\left(y_{t}-\mu_{0}\right)}^{\prime \prime }\Sigma_{\mathrm{tn}}^{-1}\left(y_{t}-\mu_{0}\right)\right]\leq w_{0}\right\}\; & \;\\ & =1-P_{\chi_{n}^{2}}(-\frac{2}{\beta }\log w_{0}),\; & \; \end{array}$$

which implies that *w*_*t*_follows $\frac{2}{\beta \times w_{0}}p_{\chi_{\left(n\right)}^{2}}(-\frac{2}{\beta }\log w_{0})$, where $\chi_{\left(n\right)}^{2}$ denotes the chi-square variable which assumes values $-\frac{2}{\beta }\log w_{0}$ for 0<*w*_0_≤1, with *n* degrees of freedom. Similarly, for DE genes (18) the *β*-weight $w_{t}=\phi_{\beta }(y_{t}|\theta ,\sigma_{t}^{2},k=1)$ also follows $\frac{2}{\beta \times w_{0}}p_{\chi_{\left(n=n_{1}+n_{2}\right)}^{2}}(-\frac{2}{\beta }\log w_{0})$, for 0<*w*_0_≤1 using the additive property of ^*χ*2^distributions.

In many cases, however, the variance is unknown. For such cases, the distribution of the *β*-weights is obtained by parametric bootstrapping. Thus statistically, we can examine whether or not a gene is contaminated by outliers using either one of the two *β*-weight functions because both weight functions follow the same distribution and show similar trends for the observed weights of both gene expression patterns (DE and EE). However, the *t*th gene is defined as contaminated by outliers if

(24) $$w_{t}=\phi_{\beta }(y_{t}|\hat{\theta },\sigma_{t}^{2},k=1)<w_{0}=\xi_{p}$$

where *ξ*_*p*_ is the *p*-quantile of the *β*-weights defined by

(25) $$\mathrm{Pr}\left\{\phi_{\beta }(y_{t}|\hat{\theta },\sigma_{t}^{2},k=1)<\xi_{p}\right\}\leq \mathrm{p.}$$

Heuristically, we choose *p*=1^0−5^ for the detection of contaminating genes. Then we compute the *β*-PPDE using equation (5) updating the measurements in the contaminated genes. To compute the *β*-PPDE with respect to a contaminating gene expression, say, for example, $y_{t}=\left(y_{t}^{\left(1\right)}:y_{t}^{\left(2\right)}\right)$ by equation (5), we modify the contaminated measurements in $y_{t}^{\left(g\right)}$ using the robust mean ${\hat{\mu }}_{\mathrm{tg}}$ obtained iteratively using equation (13). Here $y_{\mathrm{ti}}^{\left(g\right)}$ is taken to be the *i*th contaminated measurement of $y_{t}^{\left(g\right)}$ in group *g*=1, 2 if

(26) $$\psi_{\beta }(y_{\mathrm{ti}}^{\left(g\right)}|{\hat{\mu }}_{\mathrm{tg}},{\hat{\sigma }}_{\mathrm{tg}}^{2})<\alpha_{p},$$

where *α*_*p*_ is the *p*-quantile of the *β*-weights defined by

(27) $$\mathrm{Pr}\left\{\psi_{\beta }(y_{\mathrm{ti}}^{\left(g\right)}|{\hat{\mu }}_{\mathrm{tg}},{\hat{\sigma }}_{\mathrm{tg}}^{2})<\alpha_{p}\right\}\leq \mathrm{p.}$$

Here $\psi_{\beta }(y_{\mathrm{ti}}^{\left(g\right)}|\mu_{\mathrm{tg}},\sigma_{\mathrm{tg}}^{2})$ is the *β*-weight function that is used to compute the robust mean and variance (14), which follows $\frac{2}{\beta \times w_{0}}p_{\chi_{\left(1\right)}^{2}}(-\frac{2}{\beta }\log w_{0})$, where $\chi_{\left(1\right)}^{2}$ denotes the chi-square variable which assumes values of $-\frac{2}{\beta }\log w_{0}$ for 0<*w*_0_≤1, with 1 degree of freedom. However, we can set an arbitrary threshold (*α*_0_=0.2 ) to detect contaminated measurements with weights that are below the threshold, because weights are close to zero for outlying/extreme observations.

### Simulated data that were used to examine the performance of the *β*-EB approach

The *β*-EB approach that we developed detected a large proportion of outliers with p-values less than 1^0−5^. In the microarray data of head and neck cancer, 1.75% of the genes were outliers; in the lung cancer data, 13.75% were outliers; and in *Arabidopsis thaliana*, 16.59% were outliers in the empirical data analysis. A detailed inspection of the outliers detected in the lung cancer data reflected misspecification of the model. To investigate the effect of outliers and model misspecification, we conducted a numerical simulation in which we compared the performance of the proposed *β*-EB approach with the t-test, linear models for microarray data (Limma) [22], SAM [17], and other EB approaches (EB-LNN, eGG [29], eLNN [29], GaGa [21]). The t-test, Limma, and SAM detect DE genes based on p-values while, the EB procedures and the *β*−EB approach detect DE genes based on posterior probabilities. Therefore, we calculated the AUC (area under the curve) and pAUC (partial area under the curve) of the ROC curves. We also compared the estimated proportion of DE genes obtained using the *β*−EB and EB approaches. This characteristic plays an important role, especially when the aim of the study is to identify the major regulatory elements that influence the expressions of a large number of genes. The EB approaches estimate the proportion of DE genes by the mean posterior probability. The *β*−EB approach estimates it by using equation (11). No reasonable procedure to calculate the proportion of DE genes for the t-test, Limma and SAM methods could be found, because, in these methods, the estimation depends on the threshold value of the p-values.

#### Simulated gene expression profiles with and without outliers

We generated 50 datasets that roughly reflect the head and neck cancer data described in empirical data analysis below. Each dataset contained measurements of 1,000 genes, and 50 out of the 1,000 genes were DE (*p*_1_=0.05). The log-transformed expression was assumed to follow normal distribution. The mean log-expression level of a gene followed a normal distribution with the mean *μ*_0_=2.0 and the variance $\tau_{0}^{2}=3.0.$ The gene-specific variance $\sigma_{t}^{2}$ of the log expression level among the genes varied from the exponential distribution with a mean of ^*σ*2^=0.10.

We considered two scenarios with different proportions of contaminating genes (10%, 20%), and two scenarios with two patterns of outliers (mild outliers: *μ*_*ti*_*″*=5*μ*_*ti*_), and (extreme outliers: *μ*_*ti*_*″*=10*μ*_*ti*_). To estimate the dependence of the performance on the sizes of the groups, we considered two more scenarios with different group sizes (moderate/large (*n*_1_=*n*_2_=30) and small (*n*_1_=*n*_2_=10)).

#### Simulated gene expression profiles from misspecified model

To show how the *β*− weight can be used for model diagnosis, we generated the expressions of each of the 1,000 genes in the dataset from their gamma distribution. The shape parameter that we obtained followed log normal distribution with the location parameter 1 and scale parameter 1. The scale parameter of the gamma distribution was set to 0.067. The LNN model was applied to this data. When the shape parameter is large, a gamma distribution can be approximated by a log normal distribution; however, when the shape parameter is small, especially when it is smaller than 1, the gamma distribution has a heavy mass near 0 and it cannot be approximated by a log normal distribution. In our simulation scenario, the proportion of transcripts with a shape parameter <1 was 0.159. We used the dataset that contained the measurements of 1,000 genes with 30 samples in each of the two groups. The measurements for 50 out of 1,000 genes were DE (*p*_1_ = 0.05). The gene-specific variance (scale) of the log expression level among genes varied from the gamma distribution.

### The empirical data

#### Head and neck cancer data

The publicly available microarray data from the study of head and neck cancer [41] was used in this study. Most head and neck cancers are squamous cell carcinomas (HNSCC), originating from the mucosal lining (epithelium) of these regions. The data consists of the expression levels of 12,625 cellular RNA transcripts in the tumor and normal tissues from 22 patients with histologically confirmed HNSCC.

#### Lung cancer data

The publicly available microarray data from the study of two types of lung cancer [42] were used in this study. Non-small cell lung cancer (NSCLC) is the most common bronchial tumor. It has been classified into two major histological subtypes, adenocarcinoma (AC) and squamous cell carcinoma (SCC). After quality assessment of 60 microarray hybridizations, the data represent the gene expression profiles of 54,675 cellular RNA transcripts in 40 AC and 18 SCC samples [42].

#### Arabidopsis thaliana expression data

The published pre-processed expression data for 22,810 probe sets on the Affymetrix Arabidopsis ATH1 (25K) array across 1,436 hybridization experiments [43] was analyzed in the present study. The data included a high-density haplotype map of the Arabidopsis Bay-0 × Sha RIL population (211 RILs), using 578 single feature polymorphism (SFP) markers. Data obtained from TAIR (The Arabidopsis Information Resource: http://www.arabidopsis.org/) included the complete genome sequence, the gene structure, and gene product information.

## Results and discussion

### Simulation results

#### Performance of the *β*-EB approach using the simulated data with and without outliers

Table 1 shows the average estimates of the proportion of DE genes (_*p*1_), area under the ROC curve (AUC) and partial area under the ROC curve (pAUC; at FPR≤ 0.2) of the eight procedures in the case of large/moderate size of groups (_*n*1_=_*n*2_=30). In the absence of outliers, the average estimates of _*p*1_ were close to the true _*p*1_=0.05 for both the classical EB-LNN and *β*-EB approaches; the AUC and pAUC were also found to be similar for the two approaches. In the presence of outliers, as noted earlier, the average estimates of _*p*1_ were close to the true _*p*1_=0.05 for the *β*-EB approach; however, the average estimates of _*p*1_were over-estimated by all the other model based EB approaches (EB-LNN, eGG, eLNN, GaGa). The model based EB approaches were very sensitive to outliers. In the case of 20% contaminated genes with extreme outliers, the pAUC became worse in general. The three EB approaches (eGG,eLNN and GaGa) had even lower pAUC values than the t-test, Limma and SAM. The pAUC of EB-LNN was a little larger then that of the other three EB-approaches, but still worse than t-test, Limma and SAM. *β*−EB gave the large value of pAUC among all procedures. We observed the same pattern in the case of small size of groups (_*n*1_=_*n*2_=10, Table 2).

**Table 1:** **The proportion of DE genes (*p***_**1**_**=0.05), AUC, and pAUC with a FPR ≤ 0.2 estimated by the t-test, Limma, SAM, and EB approaches (EB-LNN, eGG, eLNN, GaGa) and the*β*-EB approach averaged over 50 simulated datasets: the case of large sample**

								
	**t**	**Limma**	**SAM**	**eGG**	**eLNN**	**GaGa**	**EB-LNN**	***β*-EB**
In absence of outliers								
*p*_1_	-	-	-	0.0488	0.0458	0.0494	0.0496	0.0482
	-	-	-	(0.0010)	(0.0009)	(0.0010)	(0.0010)	(0.0013)
AUC	0.9861	0.9861	0.9862	0.9848	0.9734	0.9879	0.9892	0.9890
	(0.0020)	(0.0021)	(0.0020)	(0.0019)	(0.0030)	(0.0017)	(0.0015)	(0.0016)
pAUC	0.1929	0.1934	0.1924	0.1925	0.1894	0.1940	0.1941	0.1940
	(0.0008)	(0.0008)	(0.0008)	(0.0008)	(0.0011)	(0.0006)	(0.0007)	(0.0007)
In presence of 10% contaminated genes with mild outliers								
_*p*1_	-	-	-	0.0807	0.1053	0.1008	0.0649	0.0504
	-	-	-	(0.0013)	(0.0012)	(0.0014)	(0.0013)	(0.0014)
AUC	0.9649	0.9661	0.9699	0.9515	0.9396	0.9524	0.9621	0.9870
	(0.0031)	(0.0030)	(0.0029)	(0.0030)	(0.0026)	(0.0052)	(0.0020)	(0.0019)
pAUC	0.1826	0.1830	0.1844	0.1696	0.1577	0.1649	0.1724	0.1924
	(0.0012)	(0.0012)	(0.0012)	(0.0012)	(0.0009)	(0.0008)	(0.0009)	(0.0008)
In presence of 10% contaminated genes with extreme outliers								
*p*_1_	-	-	-	0.0834	0.1076	0.1043	0.0599	0.0489
	-	-	-	(0.0015)	(0.0012)	(0.0014)	(0.0013)	(0.0014)
AUC	0.9692	0.9695	0.9676	0.9488	0.9333	0.9422	0.9601	0.9880
	(0.0031)	(0.0031)	(0.0028)	(0.0034)	(0.0030)	(0.0064)	(0.0019)	(0.0017)
pAUC	0.1842	0.1844	0.1834	0.1684	0.1542	0.1610	0.1617	0.1931
	(0.0012)	(0.0012)	(0.0011)	(0.0010)	(0.0010)	(0.0009)	(0.0010)	(0.0007)
In presence of 20% contaminated genes with mild outliers								
_*p*1_	-	-	-	0.1275	0.1693	0.1565	0.0946	0.0521
	-	-	-	(0.0016)	(0.0014)	(0.0016)	(0.0018)	(0.0016)
AUC	0.9405	0.9415	0.9430	0.9147	0.8984	0.9085	0.9502	0.9850
	(0.0041)	(0.0041)	(0.0030)	(0.0028)	(0.0025)	(0.0026)	(0.0021)	(0.0017)
pAUC	0.1728	0.1727	0.1723	0.1409	0.1214	0.1320	0.1601	0.1904
	(0.0014)	(0.0014)	(0.0011)	(0.0009)	(0.0007)	(0.0006)	(0.0014)	(0.0007)
In presence of 20% contaminated genes with extreme outliers								
_*p*1_	-	-	-	0.1260	0.1735	0.1614	0.0869	0.0502
	-	-	-	(0.0023)	(0.0014)	(0.0015)	(0.0015)	(0.0014)
AUC	0.9465	0.9460	0.9455	0.9112	0.8910	0.8980	0.9421	0.9869
	(0.0040)	(0.0040)	(0.0034)	(0.0035)	(0.0034)	(0.0035)	(0.0028)	(0.0017)
pAUC	0.1733	0.1721	0.1720	0.1391	0.117	0.1282	0.1539	0.1923
	(0.0014)	(0.0014)	(0.0012)	(0.0012)	(0.0010)	(0.0009)	(0.0016)	(0.0008)

The numbers in parentheses are the standard errors for the 50 simulation trails.

**Table 2:** **The proportion of DE genes (*p***_**1**_**=0.05), AUC, and pAUC with a FPR ≤ 0.2 estimated by the t-test, Limma, SAM, and EB approaches (EB-LNN, eGG, eLNN, GaGa) and the*β*-EB approach averaged over 50 simulated datasets: the case of small sample**

								
	**t**	**Limma**	**SAM**	**eGG**	**eLNN**	**GaGa**	**EB-LNN**	***β*-EB**
In absence of outliers								
_*p*1_	-	-	-	0.0489	0.0430	0.0482	0.0502	0.0518
	-	-	-	(0.0010)	(0.0009)	(0.0009)	(0.0009)	(0.0009)
AUC	0.9688	0.9707	0.9675	0.9721	0.9614	0.9780	0.9780	0.9781
	(0.0026)	(0.0023)	(0.0023)	(0.0023)	(0.0023)	(0.0016)	(0.0016)	(0.0016)
pAUC	0.1858	0.1865	0.1849	0.1858	0.1839	0.1873	0.1870	0.1872
	(0.0009)	(0.0008)	(0.0008)	(0.0007)	(0.0009)	(0.0007)	(0.0007)	(0.0007)
*p*_1_	-	-	-	0.0936	0.1153	0.1106	0.0451	0.0529
	-	-	-	(0.0013)	(0.0010)	(0.0012)	(0.0010)	(0.0009)
AUC	0.9466	0.9487	0.9452	0.9352	0.9235	0.9444	0.9626	0.9740
	(0.0030)	(0.0028)	(0.0030)	(0.0027)	(0.0025)	(0.0020)	(0.0018)	(0.0017)
pAUC	0.1773	0.1766	0.1733	0.1591	0.1477	0.1595	0.1769	0.1839
	(0.0010)	(0.0011)	(0.0009)	(0.0011)	(0.0009)	(0.0009)	(0.0008)	(0.0008)
In presence of 10% contaminated genes with extreme outliers								
*p*_1_	-	-	-	0.0919	0.1210	0.1167	0.0379	0.0523
	-	-	-	(0.0011)	(0.0010)	(0.0011)	(0.0009)	(0.0009)
AUC	0.9399	0.9418	0.9439	0.9347	0.9145	0.9344	0.9447	0.9766
	(0.0036)	(0.0035)	(0.0034)	(0.0024)	(0.0029)	(0.0020)	(0.0025)	(0.0016)
pAUC	0.1740	0.1716	0.1710	0.1569	0.1413	0.1512	0.1668	0.1859
	(0.0011)	(0.0012)	(0.0012)	(0.0009)	(0.0009)	(0.0008)	(0.0011)	(0.0007)
In presence of 20% contaminated genes with mild outliers								
*p*_1_	-	-	-	0.1398	0.1883	0.1725	0.0435	0.0522
	-	-	-	(0.0016)	(0.0011)	(0.0013)	(0.0010)	(0.0009)
AUC	0.9208	0.9213	0.9214	0.9049	0.8825	0.9099	0.9301	0.9710
	(0.0035)	(0.0034)	(0.0035)	(0.0027)	(0.0030)	(0.0024)	(0.0022)	(0.0018)
pAUC	0.1678	0.1617	0.1595	0.1335	0.1120	0.1304	0.1510	0.1818
	(0.0011)	(0.0014)	(0.0013)	(0.0012)	(0.0011)	(0.0011)	(0.00126)	(0.0009)
In presence of 20% contaminated genes with extreme outliers								
_*p*1_	-	-	-	0.1380	0.2001	0.1832	0.0343	0.0535
	-	-	-	(0.0029)	(0.0011)	(0.0012)	(0.0009)	(0.0009)
AUC	0.9103	0.9109	0.9162	0.8877	0.8680	0.8914	0.9122	0.9753
	(0.0043)	(0.0041)	(0.0040)	(0.0031)	(0.0032)	(0.0027)	(0.0032)	(0.0016)
pAUC	0.1633	0.1561	0.1565	0.1195	0.1018	0.1163	0.1434	0.1840
	(0.0013)	(0.0015)	(0.0013)	(0.0017)	(0.0010)	(0.0010)	(0.0015)	(0.0008)

The numbers in parentheses are the standard errors for the 50 simulation trails.

The *β*-weights in the *β*-EB approach can be used not only to detect outliers, but also to diagnose the model assumptions. When the *β*-weights for each gene in the simulation data were calculated, the predictive distribution reflected the observed distribution and outliers with unstable expressions were identified by their low weights with p-values <1^0−5^ (see the Additional file 1: Figure S1).

In the absence of outliers, *β*was selected to be 0 for more than half the cases, while in the presence of outliers, *β*was selected to be 0.015 on average. When outliers were present, there were no cases where the *β* was selected to be 0. This result implies that the selected value of *β*could be used as a predictor of the presence of outliers.

#### The use of the *β*− weight to diagnose model misspecification

**Figure 1:**
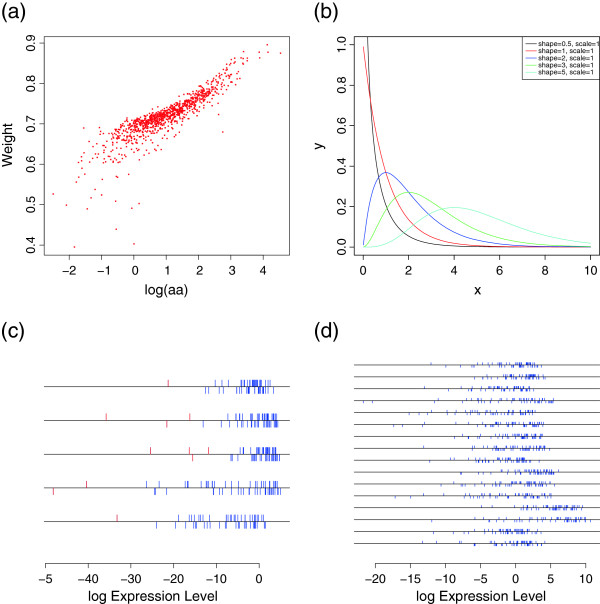
***β*-weights can diagnose a misspecified model.** (**a**) Scatter plot of log(aa) versus *β*-weight. Many of the genes with a shape parameter (aa) less than 1 have small *β*− weights. (**b**) The true distribution of gamma for different values of the shape parameter when the value of scale parameter is one. (**c**) The log-transformed expressions based on genes between weight < 0.53 and log(aa) < -1 in (a) are plotted below the lines for group 2 tissues and above the lines for group 1 tissues. The genes with low *β*− weights were shown to have heavy lower tails. (**d**) The log-transformed expressions based on genes between weight ≥ 0.6 and log(aa) < -1 in (a) are plotted below the lines for group 2 tissues and above the lines for group 1 tissues. The log-transformed expression profiles of these genes were shown to be similar to the normal distribution.

To investigate the use of the *β*− weight as a sensor for model diagnosis, we generated the expressions of each gene in the simulated data set from their gamma distribution. Many of the genes with shape parameters (aa) less than 1 have small *β*− weights (Figure 1(a)). The gamma distribution with aa<1 has a high probability of being close to 0 Figure 1(b), and cannot be approximated by the log normal distribution. Genes with low *β*− weights are found to have heavy lower tails (Figure 1(c)). Some genes, however, with aa<1 have moderate *β*− weights and the log-transformed expression profiles of these genes were similar to the normal distribution (Figure 1(d)). To see the performance for the case of model mis-specification, we compared our method with EB-LNN approach. We showed the average estimates of the proportion of DE genes (_*p*1_), mis-specification rates (MR), false positive rates (FPR), false negative rates (FNR) by controlling false discovery rate (FDR) at 0.01. We also compared pAUC (at FPR≤ 0.2). The current modification of outliers did not rescue the effect of model misspecification well regarding with the detection of DE genes (Table 3). Currently, the information is equally treated among transcripts when DE transcripts are identified. That is, the identification of DE transcripts depends on the ratio of _*f*1_ and _*f*0_and does not depend on the absolute values. When these values are very small, we may suspect that the expression profile of the transcript is not consistent with the specified model and may postpone the solid decision. The improved procedure will discount the information content of transcripts with low *β*-weight. On the other hand, the bias of the estimated proportion of DE genes _*p*1_ was reduced in the *β*−EB approach. This is because the estimation of _*p*1_puts different weight among transcripts (Equation 10).

**Table 3:** **The proportion of DE genes (**_***p*1**_**=0.05), MR, FPR, FNR with controlled value of FDR at 0.01, and pAUC (at FPR ≤ 0.2) for EB and*β*-EB approaches averaged over the 50 simulated datasets from the gamma distribution**

					
	**p**	**MR**	**FPR**	**FNR**	**pAUC**
In the case of model mis-specification					
EB-LNN	0.0309	0.0287	0.0002	0.5776	0.1359
	(0.00054)	(0.0004)	(0.00004)	(0.0081)	(0.0013)
*β*-EB	0.0371	0.0281	0.0002	0.5704	0.1361
	(0.0006)	(0.00038)	(0.00004)	(0.008)	(0.0014)

The genes with the posterior probabilities of DE ≥ 0.674 for EB-LNN and posterior probabilities of DE ≥ 0.902 for *β*−EB by controlling FDR at 0.01. The numbers in parentheses are the standard errors for the 50 simulation trails.

### Analysis of the head and neck cancer data

**Figure 2:**
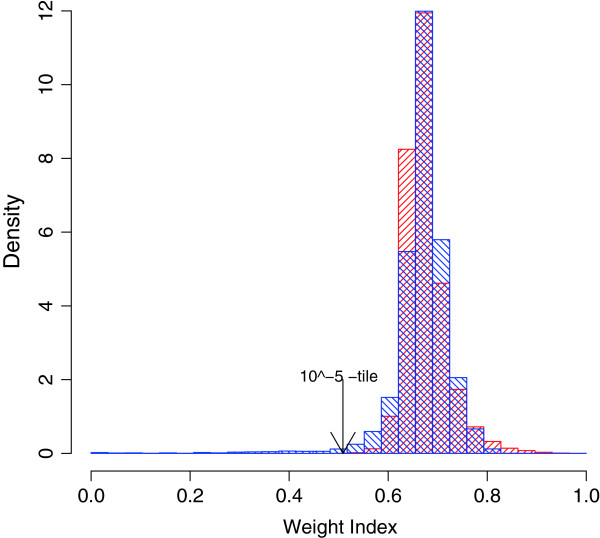
**The distribution of the*β*weights for the head and neck cancer data.** The observed distribution (blue) of *β*-weights was qualitatively similar to the parametric bootstrap-based predicted distribution (red) with the exception of 261 outliers (2.2% of the total genes) with small *β*-weights (*p*<10^5^).

**Figure 3:**
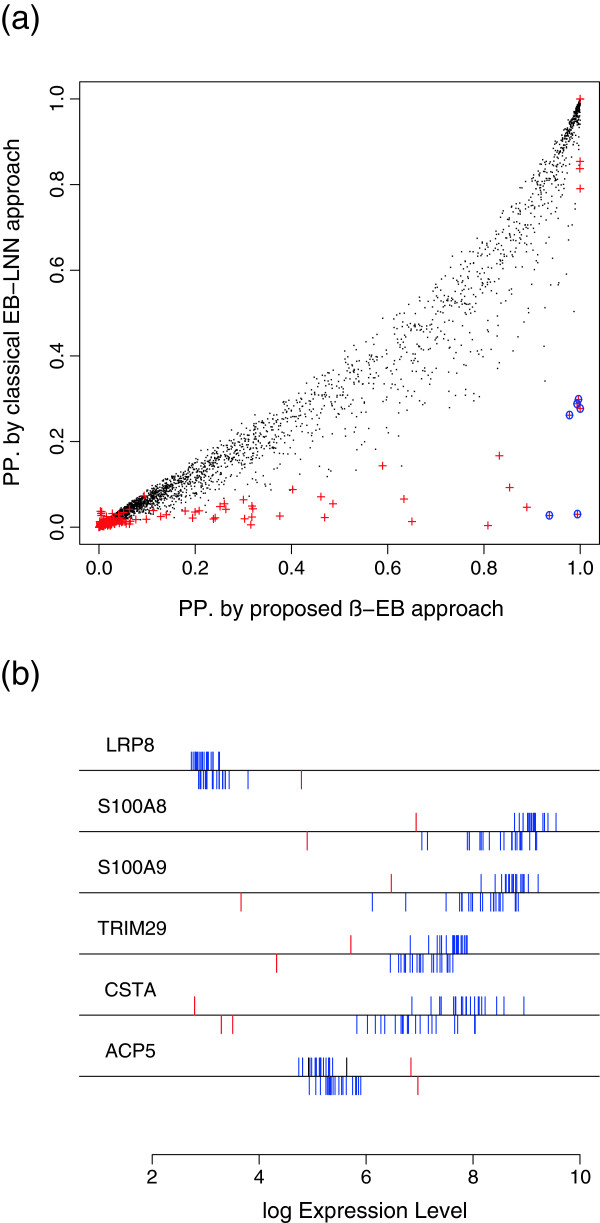
**Posterior probabilities estimated by EB and*β*-EB for the head and neck cancer data.** (**a**) Scatter plot of the posterior probabilities (pp.) estimated by the proposed *β*-EB approach and by the classical EB-LNN approach. The red “+” marks represent outliers with *β*-weights for which the p-values <10^−5^. The blue “o” marks the outliers that were identified as DE by the *β*-EB approach (*pp.*>0.95) and as EE by the original EB approach (*pp.*<0.5). (**b**) Expression levels of the six genes (marked by the blue “o” in (a)) that were identified as DE by the *β*-EB approach and as EE by the EB approach. The log-transformed expressions are plotted below the lines for the tumor tissues and above the lines for the normal tissues. Outliers with low *β*-weights are indicated in red.

Assuming the LNN model, we used the *β*-EB approach to analyze the head and neck cancer data [41]. By cross-validation, the tuning parameter *β*was estimated to be 0.016 [see Additional file 1: Figure S2(a)]. The distribution of *β*-weights was qualitatively similar to the previously reported parametric bootstrap-based predictive distribution for all but 261 outliers (2.2% of the total genes) that have small *β*-weights for which *p*<1^0−5^ (Figure 2). Because the sample size was large, the EB and *β*-EB approaches both generated consistently decisive results for the proportion of DE/EE for most of the genes. Of the 12,625 genes, 9,538 were estimated to be EE with posterior probabilities >0.95 (posterior probabilities of DE were <0.05). Both methods estimated the same 525 genes to be DE with posterior probabilities >0.95 (Figure 3(a)). The mixing proportion of the DE genes *p*_1_ for the classical EB-LNN and *β*-EB approaches was estimated to be 0.095 and 0.084 respectively. The classical EB-LNN approach may have overestimated the proportion of DE genes (see Table 1).

The *β*-EB approach detected six contaminating genes (LRP8, S100A8, S100A9, TRIM29, CSTA, ACP5) as outliers with the posterior probability of DE >0.95; the posterior probability for these genes by the classical EB-LNN approach was <0.5. For the most part, even after log transformation, these genes were over-expressed or under-expressed in only one or two of the samples (Figure 3(b)). There is strong evidence that links all of these genes with cancer.

Aberrations of the short arm of chromosome 1 (1p) are common events in lung and many other types of cancer. The low-density lipoprotein receptor-related protein 8 (LRP8) which is associated with the Wnt developmental pathway is coded by a gene on chromosome 1p; this gene has been shown to be over-expressed in lung cancer [44]. Wnt ligands bind to LRPs, and interfere with the multi-protein APC/*β*-catenin destruction complex. The complex role of *β*-catenin in cell proliferation and cell adhesion has been the main focus of many mechanistic studies.

S100 proteins, belonging to the superfamily of EF-hand calcium-binding proteins, are involved in cellular processes translating changes in Ca^2^+ levels into specific cellular responses by binding to target proteins. At least 16 genes of the multigenic S100 family, including the genes coding for S100A8 (MRP8 or calgranulin A) and S100A9 (MRP14 or calgranulin B), are clustered on human chromosome 1q21, a region that is a frequent target for the chromosomal rearrangements that occur during tumor development. The complex of S100A8 and S100A9 (also called calprotectin) is actively secreted during the stress response of phagocytes [45]. The complex activates the signaling pathways that promote tumor growth and metastasis by inducing the expression of multiple downstream protumorigenic effector proteins [46]. The classical EB-LNN approach strongly identified S100A8 and S100A9 as EE genes with posterior probabilities of DE being 0.027 and 0.030 respectively.

The TRIM29 protein (tripartite motif-containing protein 29) was reported to bind p53 and antagonize p53-mediated functions [47]. CSTA (stefin-A) inhibits the cysteine proteinases that participate in the dissolution and remodeling of connective tissue and basement membranes in the processes of tumor growth, invasion, and metastasis [48]. Tartrate-resistant acid phosphatase 5 (ACP5 or TRAP) may act as a growth factor to promote proliferation and differentiation of osteoblastic cells and adipocytes. The intensity of histochemical activity in several human breast cancer cell lines and tissues that express TRAP was found to correlate with the degree of tumorigenicity [49].

The classical EB-LNN approach attached lower posterior probabilities to these genes, probably because the extraordinary expression of these genes in a few samples led to an over-estimation of the variances within the groups.

### Analysis of the lung cancer data

**Figure 4:**
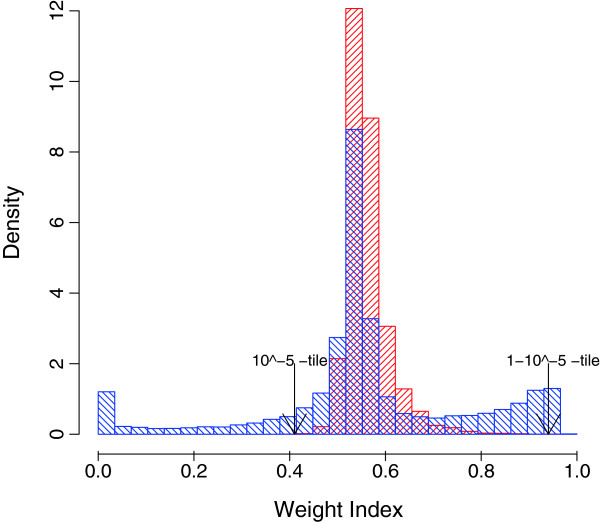
**The distribution of the*β*weights for the lung cancer data.** The observed distribution (blue) of *β*-weights showed a large deviation from the predicted distribution (red). Because the observed distribution has extremely heavy tails on both sides compared with the predicted distribution, we put lower and upper 1^0−5^tiles for the predicted distribution.

**Figure 5:**
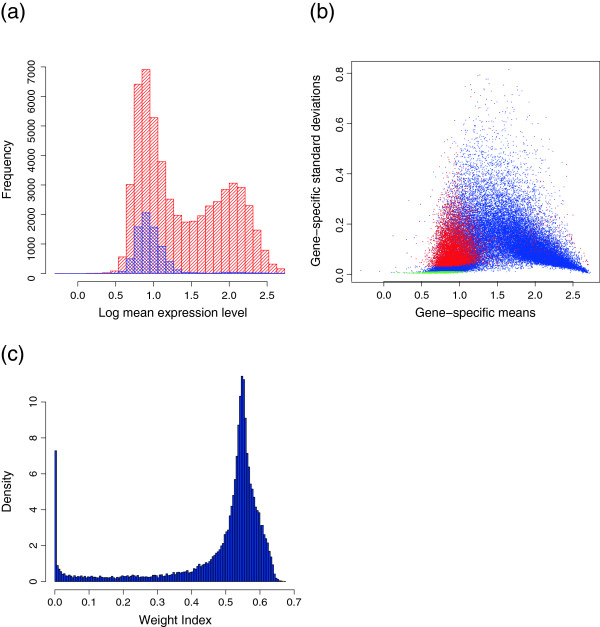
**Features of the expression profiles of the two types of lung cancer data.(a)** Distribution of the log mean expression levels. The distribution of the outlier genes is shown distribution in blue. **(b)** Scatter plot of gene-specific means versus standard deviations. The red dots represent genes with low *β*-weights (*p*<1^0−5^); green dots represent genes with high weights (*p*<1^0−5^); and the blue dots represent the outlier genes. **(c)** When transcripts with little variation (standard deviation < 0.05) were excluded, the upper heavy tail observed in Figure 4 disappeared.

The value of *β*was estimated to be 0.018 (Additional file 1: Figure S2(b)). The *β*-weight distribution of the two types of lung cancer data [42] showed a large deviation from the predicted distribution (Figure 4). The *β*-weight distribution had heavy tails on both sides, suggesting that some of the assumptions behind the LNN model were violated. We inspected the distribution of the mean expression levels of the genes and found that the distribution of the mean log-transformed expression levels is bi-modal and not uni-modal (Figure 5(a)). Most of the genes that had unexpectedly low and unexpectedly high weights had low mean-expression levels. To further investigate the properties of the outliers, we plotted the standard deviations against the means of the log-transformed expression levels of the genes (Figure 5(b)). We found that the genes with extremely low weights tended to have large standard deviations, implying their irregular expression in some samples. Genes with extremely high weights had low standard deviations and low means.

The *β*-weight is a monotone decreasing function of the squared Mahalanobis Distance between the log transformed expression profile and the transcript specific log transformed mean (equations 17 and 18). When the transcripts with little variation (standard deviation < 0.05) were excluded, the upper heavy tail disappeared (Figure 5(c)).

### Analysis of the *Arabidopsis thaliana* microarray data

**Figure 6:**
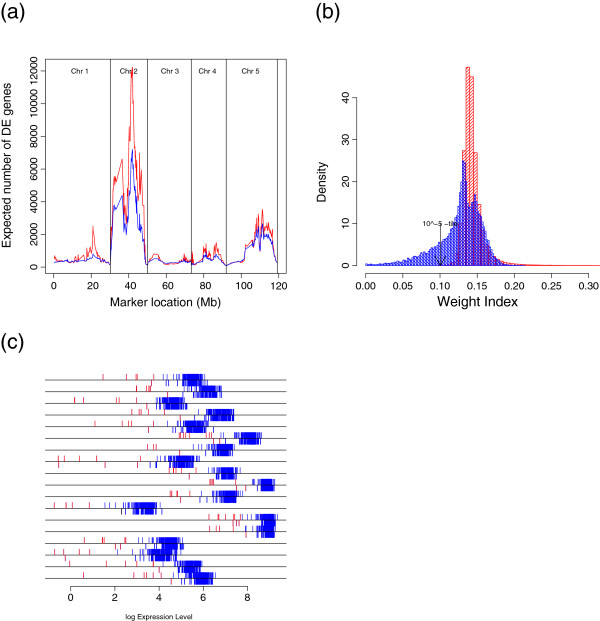
**Genomic architecture of the eQTL study across the five*A. thaliana*chromosomes.** (**a**) Expected numbers of DE transcripts/e-traits (y-axis) plotted against the marker location in mega bases (Mb) on the x-axis. (**b**) Parametric predicted distribution (red) and observed distribution (blue) of *β*-weights for the *A. thaliana* data were measured for marker 73 on chromosome 4. The observed distribution showed a large deviation from the predicted distribution. (**c**) Expression levels of the 18 transcript with weights less than 0.003 (i.e., w < .003). The log-transformed expressions are plotted below the lines for marker genotype “B” and above the lines or marker genotype “A”. Outliers with low *β*-weights are indicated in red.

Assuming the LNN model, we applied the proposed *β*-EB approach to the combined microarray data and marker genotypes information from *A. thaliana*. To identify transcripts that are significantly linked to genomic locations, at each marker we tested for significant linkage across transcripts instead of testing each transcript for significant linkage across markers. This procedure amounted to identifying DE transcripts at each marker, with groups determined by marker genotypes “A” and “B”. For simplicity, we considered a backcross population from two inbred parental populations, P1 and P2, genotyped as either A or B at the M markers. The *β*-EB approach predicted a large number of DE genes compared with the classical EB-LNN approach, because of some gene expressions breakdown the normality assumptions or contaminated by outliers (Figure 6(c)). Through cross-validation, the tuning parameter *β*was estimated to be 0.016 for chromosomes 1-5. Here, we focus on a telomeric region of chromosome 4, where *β*-EB detected potential hotspots and the classical EB-LNN did not (Figure 6(a)). The parametric predicted distribution and observed distribution of the weights of the data from *A. thaliana* were measured for marker 73 on chromosome 4. The *β*-weight distribution showed a large deviation from the predicted distribution (Figure 6(b)). The expression levels of the 18 transcript with weights less than 0.003 (i.e., w < .003) are shown in Figure 6(c). The log-transformed expressions at marker genotype B are plotted below the lines while those at marker genotype A are plotted above the lines. Outliers with low weights are in red. According to information obtained from the Arabidopsis gene regulatory information server (AGRIS) [50], this region inclu des three transcription factors one of which is CYC1 (cyclin-dependent protein kinase regulator) [51].

## Conclusions

The microarray technique has opened the door to the study of the transcriptome. The methods used to analyze microarray data can also be applied to expression proteomics data which measures the end product of the gene expression cascade, the mature protein, and is more closely related to the biological function than data at the message levels [52]. To analyze these data it is essential to be able to detect genes or proteins that are DE under different conditions or environments. Parametric models are useful for the efficiency of the estimation and also for the biological interpretation of the outputs. In this study, we observed that standard likelihood approaches, or Bayesian approaches that are based on likelihoods, may misidentify some crucial genes in test data sets from cancer studies. Whether or not the observed abnormal expressions are unique to the gene expressions in cancer tissues or whether this is present even in normal tissues where the irregular expressions of genes may be found under stress conditions is still unclear. However, the two examples of microarray gene expression data that we examined in this study imply that it is difficult to develop a single parametric model that effectively describes microarray data in all cases. Several statistical approaches for the identification of DE genes have been developed. However, the accuracy of most of them suffer when contaminating genes or irregular patterns of expressions are present. A few robust algorithms for the identification of DE genes are available. However, these algorithms do not address the problem of the identification of contaminating genes. It is, therefore, difficult to scrutinize or diagnosis the contaminating DE genes from a reduced gene expression data set and further statistical investigations, like clustering/classification, using reduced gene expression datasets containing contaminating DE genes may produce misleading results.

In this paper, we describe the *β*-EB procedure that we have developed. This procedure extends the EB-LNN model using *β*-divergence. To overcome the problems mentioned above, this *β*-EB approach assumes gene-specific variance. We estimated the model parameters by maximizing the *β*-likelihood function using an EM-like algorithm. The gene-specific variance was estimated separately outside the EM algorithm. To avoid the overestimation of gene-specific variance, we adopted the *β*-likelihood approach for each gene, with the value of *β*set to 0.1 based on the result of an earlier study [39]. Then, the posterior probability of differential expression and *β*-weights for identification of DE genes and contaminating genes, respectively, are computed. The values of the *β*-weights are between 0 and 1. Contaminating genes are defined as having the smaller *β*-weights. In addition, we discuss the statistical significance of contamination using the distribution of *β*-weights. The contaminated expressions are updated by a robust group mean [39] and the posterior probability of differential expression of contaminating genes are updated using the previous estimates of the model parameters. Thus, our method does not sacrifice computational efficiency. The proposed method can be used to improve the results of further statistical investigations like clustering/classification when reduced gene expression datasets are used.

While the proposed *β*-EB procedure preserves the merits of parametric hierarchical models, it is also highly robust against outliers. The value of the tuning parameter *β*plays an important role in the performance of the proposed method. The *β* parameter is selected using cross-validation. The idea of *β*-weights that we have used here can be applied to any other likelihood based statistical model for diagnosis and may prove to be a useful tool for transcriptome and proteome studies.

## Availability and requirements

The R code is available in the Additional file 2.**Contact:** mollah@lbm.ab.a.u-tokyo.ac.jp

## Competing interests

The authors declare that they have no competing interests.

## Authors’ contributions

MMHM, MNHM and HK worked together to develop the new statistical procedure. MMHM conducted the gene expression data analysis. MMHM drafted, and HK and MNHM finalized the manuscript. All authors read and approved the final version of the manuscript.

## Supplementary Material

Additional file 1 **Figure S1.** An example of a SparSNP workflow, covering basic quality control, training the model on discovery data, applying the model to validation data, plotting the results, and post-processing. **Figure S2.** Selection of the tuning parameter *β*by cross validation. (a) Selection of *β*by cross validation for head and neck cancer data. (b) Selection of *β*by cross validation for lung cancer data. Click here for file

Additional file 2 **The R-code that was used in the analysis.** Details of the implementation of SparSNP and other supplementary results. Click here for file
