# Supplementary material for: β-empirical Bayes inference and model diagnosis of microarray data
Source: BMC Bioinformatics. 2012 Jun 19;13:135. doi: 10.1186/1471-2105-13-135 (PMC3464654; doi:10.1186/1471-2105-13-135)
Supplement: Additional file 1 — Figure S1. An example of a SparSNP workflow, covering basic quality control, training the model on discovery data, applying the model to validation data, plotting the results, and post-processing. Figure S2. Selection of the tuning parameter βby cross validation. (a) Selection of βby cross validation for head and neck cancer data. (b) Selection of βby cross validation for lung cancer data. [file 1471-2105-13-135-S1.pdf]

β-empirical Bayes inference and model diagnosis of microarray data (Supplementary information)

Md. Manir Hossain Mollah, Md. Nurul Haque Mollah and Hirohisa Kishino

(a)

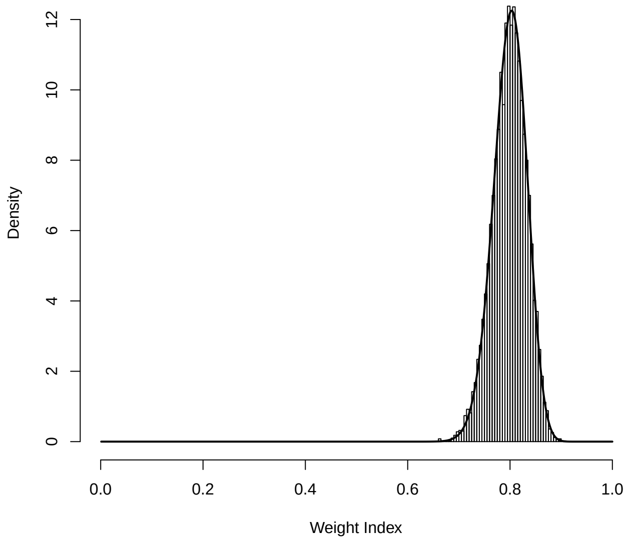

(b)

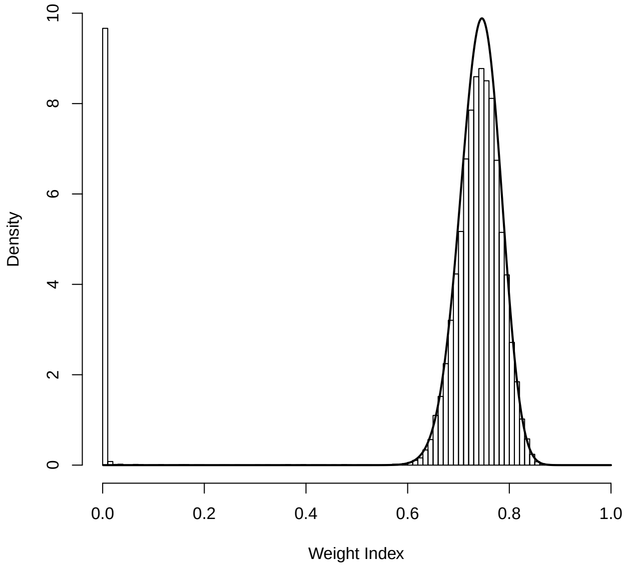

Figure S1: Predicted and observed distribution of weights by the simulation of microarray data. (a) predicted and observed distribution of weights by the simulation of microarray data without outliers. (b) predicted and observed distribution of weights by the simulation of microarray data with outliers.

(a)

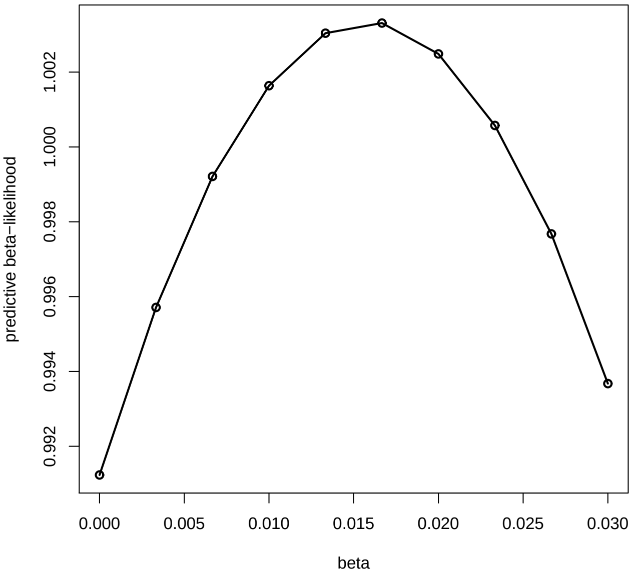

(b)

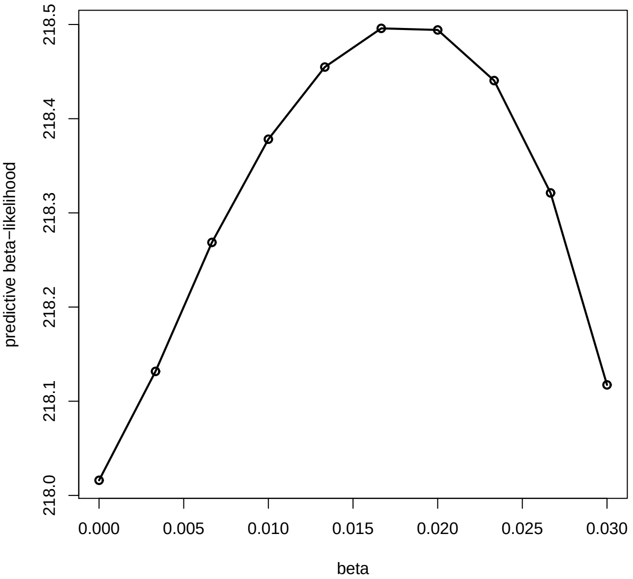

Figure S 2: Selection of the tuning parameter β by cross validation. (a) Selection of β by cross validation for head and neck cancer data. (b) Selection of β by cross validation for lung cancer data.
